# Transglutaminase 2 Undergoes a Large Conformational Change upon Activation 

**DOI:** 10.1371/journal.pbio.0050327

**Published:** 2007-12-18

**Authors:** Daniel M Pinkas, Pavel Strop, Axel T Brunger, Chaitan Khosla

**Affiliations:** 1 Department of Chemical Engineering, Stanford University, Stanford, California, United States of America; 2 Howard Hughes Medical Institute, Stanford University, Stanford, California, United States of America; 3 Department of Molecular and Cellular Physiology, Stanford University, Stanford, California, United States of America; 4 Department of Neurology and Neurological Sciences, Stanford University, Stanford, California, United States of America; 5 Department of Structural Biology, Stanford University, Stanford, California, United States of America; 6 Department of Photon Science, Stanford University, Stanford, California, United States of America; 7 Department of Chemistry, Stanford University, Stanford, California, United States of America; 8 Department of Biochemistry, Stanford University, Stanford, California, United States of America; University of Michigan, United States of America

## Abstract

Human transglutaminase 2 (TG2), a member of a large family of enzymes that catalyze protein crosslinking, plays an important role in the extracellular matrix biology of many tissues and is implicated in the gluten-induced pathogenesis of celiac sprue. Although vertebrate transglutaminases have been studied extensively, thus far all structurally characterized members of this family have been crystallized in conformations with inaccessible active sites. We have trapped human TG2 in complex with an inhibitor that mimics inflammatory gluten peptide substrates and have solved, at 2-Å resolution, its x-ray crystal structure. The inhibitor stabilizes TG2 in an extended conformation that is dramatically different from earlier transglutaminase structures. The active site is exposed, revealing that catalysis takes place in a tunnel, bridged by two tryptophan residues that separate acyl-donor from acyl-acceptor and stabilize the tetrahedral reaction intermediates. Site-directed mutagenesis was used to investigate the acyl-acceptor side of the tunnel, yielding mutants with a marked increase in preference for hydrolysis over transamidation. By providing the ability to visualize this activated conformer, our results create a foundation for understanding the catalytic as well as the non-catalytic roles of TG2 in biology, and for dissecting the process by which the autoantibody response to TG2 is induced in celiac sprue patients.

## Author Summary

The transglutaminase family of enzymes is best known for crosslinking proteins to form networks that strengthen tissues. Although this enzyme family has been extensively studied, a detailed understanding of the catalytic mechanism has been hampered by the lack of a structure in which the enzyme is active. We have solved, at atomic resolution, the structure of transglutaminase 2 (TG2) in complex with a molecule that mimics a natural substrate. The structure exposes the active site, giving direct insights into the catalytic mechanism. Unexpectedly, we observed a very large conformational change with respect to previous transglutaminase structures. Very few proteins have been observed to undergo this type of large-scale transformation. We propose a role for this structural rearrangement in the early stages of celiac disease, an autoimmune disorder in which TG2 is the principal autoantigen. Besides the fundamental implications, our results should allow for the rational design of better inhibitors of TG2 for pharmacological and therapeutic purposes.

## Abstract 3

By using a chemical biological approach, the authors observed a 12-nanometer conformational change in this ubiquitous and multifunctional protein, revealing its active site. Fundamental, pathological, and pharmacological implications are discussed.

## Introduction

Transglutaminases play important roles in diverse biological functions by selectively crosslinking proteins. They catalyze, in a Ca^2+^-dependent manner, the transamidation of glutamine residues to lysine residues, resulting in proteolytically resistant N^ɛ^(γ-glutamyl)lysyl isopeptide bonds [1–3]. The resulting crosslinked protein structures add strength to tissues and increase their resistance to chemical and proteolytic degradation. Among the members of this enzyme family are factor XIIIa, the subunit of plasma transglutaminase that stabilizes fibrin clots; keratinocyte transglutaminase, and epidermal transglutaminase, which crosslink proteins on the outer surface of the squamous epithelium [4]; and transglutaminase 2, the ubiquitous transglutaminase that is the subject of our study.

**Figure 1:**
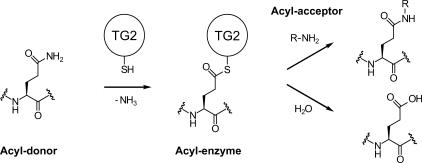
Reactions Catalyzed by TG2 TG2 can catalyze the transamidation of Gln to a suitable amine or the deamidation of Gln to Glu.

Transglutaminase 2 (TG2, also known as tissue transglutaminase) is structurally and mechanistically complex, and has both intracellular and extracellular functions [1,5]. The catalytic mechanism, related to that of cysteine proteases, involves an active site thiol that reacts with a glutamine side chain of a protein or peptide substrate to form a thioester intermediate from which the acyl group is transferred to an amine substrate. In the absence of a suitable amine, water can act as an alternative nucleophile, leading to deamidation of the glutamine residue to glutamate (Figure 1) [6]. Its catalytic activity requires millimolar Ca^2+^ concentrations and is inhibited by guanine nucleotides. Thus, intracellular TG2 lacks enzyme activity; instead, it functions as a G-protein in the phospholipase C signal transduction cascade [7]. Outside the cell, TG2 shapes the extracellular matrix by binding tightly to both fibronectin in the extracellular matrix and integrins on the cell surface [8,9] and promotes cell adhesion, motility, signaling, and differentiation in a manner independent of its catalytic activity [9–11]. Despite the variety of functions in which TG2 acts, knockout mice are anatomically, developmentally, and reproductively normal [12,13].

Although the x-ray crystal structures of several transglutaminases (including human TG2) have been solved [14–17], in each case the protein has been crystallized in a state in which the active site is obscured. Here, we report the x-ray crystal structure of human TG2 in a fundamentally novel conformation with the active site exposed. Solving this structure required stabilization of a transient state of a gluten peptide–enzyme complex using a chemical biology approach. Together with structure-based mutagenesis and related biochemical experiments, the new TG2 structure provides direct mechanistic insights into isopeptide bond formation by TG2. As the prototypical x-ray crystal structure of a catalytically activated transglutaminase, it also provides a fundamentally new opportunity to evaluate the chemistry, biology, and evolution of this remarkable protein family, as well as the role of TG2 in various pathogenic processes.

## Results

### Trapping a Transient Intermediate of TG2-Catalyzed Transamidation

**Figure 2:**
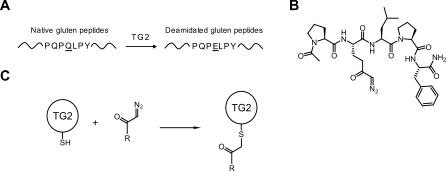
Inactivation of TG2 by a Reactive Gluten Peptide Mimic (A) In the pathogenesis of celiac sprue, TG2 deamidates specific Gln residues in gluten peptides to Glu. (B) The inhibitor Ac-P(DON)LPF-NH_2_ mimics a gluten peptide sequence that has high affinity for TG2. DON is the electrophilic amino acid 6-diazo-5-oxo norleucine. (C) The active-site Cys residue of TG2 nucleophilically attacks DON, resulting in a stable thioether adduct.

It has been suggested that TG2–gluten peptide complexes play a role in the development of anti-TG2 autoantibodies in the pathogenesis of celiac sprue, an autoimmune disorder induced by the ingestion of gluten in genetically susceptible individuals [18]. To investigate this hypothesis, we synthesized a pentapeptide Ac-P(DON)LPF-NH_2_, where DON is the electrophilic amino acid 6-diazo-5-oxo-L-norleucine (Figure 2). Its natural homolog PQLPY is found repeatedly in the sequences of gluten proteins, where it undergoes TG2-catalyzed deamidation prior to binding to the MHC class II immune receptor human leukocyte antigen (HLA)-DQ2, causing T cell proliferation in celiac sprue patients [18]. Ac-P(DON)LPF-NH_2_ is an exceptionally high-affinity irreversible inhibitor of human TG2 with inhibition parameters K_i_ = 60 nM and k_i_ = 0.5 min^−1^. Its attachment to the active-site Cys residue via a thioether linkage was verified by trypsinolysis followed by LC/MS (unpublished data). The TG2-peptide adduct is remarkably stable, which facilitated repurification and crystallization.

### Structure of the TG2-Inhibitor Complex

**Figure 3:**
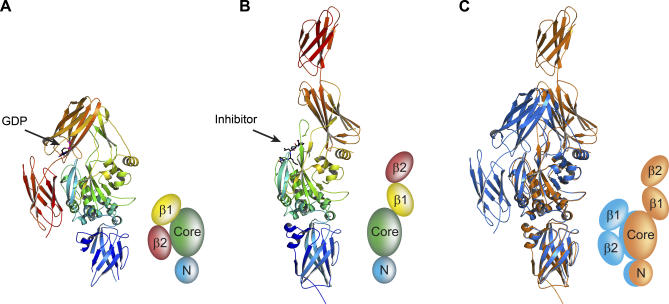
Overall Structures of GDP-Bound and Inhibitor-Bound TG2 The crystal structures are shown as ribbons, and simplified cartoons are included for clarity. (A and B) The N-terminal β-sandwich is shown in blue (N), the catalytic domain (Core) in green, and the C-terminal β-barrels (β1 and β2) in yellow and red, respectively. (A) GDP-bound TG2 [16]. (B) TG2 inhibited with the active-site inhibitor Ac-P(DON)LPF-NH_2_. (C) The N-terminal β-sandwich and catalytic domains of the two structures are superimposed, highlighting the conformational change. The GDP-bound structure is shown in blue and the inhibitor-bound structure in gold.

Human TG2 consists of four domains: an N-terminal β-sandwich (which, in contrast to previously solved TG2 structures, is fully resolved here) that binds to fibronectin; the catalytic domain containing a Cys-His-Asp catalytic triad; and two C-terminal β-barrels belonging to the fibronectin Type III CATH superfamily [16]. However, in contrast to an earlier structure, which showed human TG2 in a “closed” GDP-bound conformation (Figure 3A), the inhibitor-bound structure is in an extended or “open” conformation (Figure 3B). When the N-terminal and catalytic domains of the two structures are superimposed, the C-terminal residues are displaced by as much as 120 Å (Figure 3C). This remarkably large conformational change is reminiscent of other multidomain proteins, including, for example, the influenza hemagglutinin trimer, which undergoes a pH-dependent conformational change [19]. The change in TG2 tertiary structure is accompanied by changes in secondary structure in the hinge region. For instance, a β-strand in the closed structure consisting of residues Leu-312 to Arg-317 becomes α-helical in the open structure, inducing backbone rearrangements that extend to the active site.

**Figure 4:**
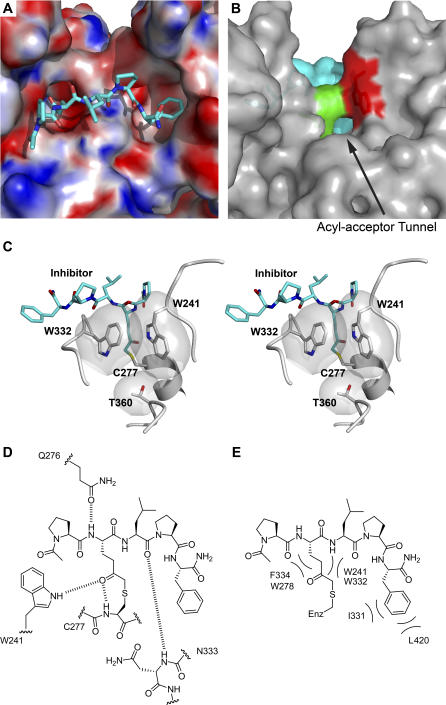
The Active Site of TG2 and Enzyme–Inhibitor Interactions (A) Electrostatic potential surface of TG2 (red indicates negative charge; blue, positive; contoured at −15 k_B_T to +15 k_B_T) in the vicinity of the peptide inhibitor. (Carbon is indicated by cyan; nitrogen by blue; and oxygen by red.) (B) Surface representation of the active-site tryptophan bridge. W332, W241, and inhibitor are shown in green, red, and cyan, respectively. The proposed acyl-acceptor approach site is indicated. (C) Stereo representation of the active site of TG2. The backbone of TG2 is shown as ribbons. The bridge tryptophans and a T360 that resides in front of the proposed acyl-acceptor entrance are shown as sticks with semitransparent surfaces. It can be seen that the bridging tryptophan residues reside on separate loops above the catalytic Cys (sulfur is indicated by yellow). The thioether attachment of the inhibitor (cyan indicates inhibitor carbons, and gray indicates TG2 carbons) is also evident. (D) Hydrogen-bonding interactions between TG2 and the peptide are shown as dashed lines. (E) Schematic diagram of hydrophobic interactions between TG2 and the inhibitor.

The catalytic Cys (C277) is located in a hydrophobic tunnel bridged by W241 and W332, which reside on separate loops in the active site (Figure 4C). The inhibitor, which mimics the acyl-donor substrate, occupies one side of the active-site tunnel (Figure 4A). The other side of the tunnel is open (Figure 4B) and presumably serves as the binding site for a lysine residue or alternative nucleophilic substrate that participates in the transamidation reaction. Notably, the inhibitor ketone is hydrogen bonded to the indole of Trp-241, as well as to the backbone amide of the catalytic cysteine (Figure 4D), analogous to the hydrogen bonding arrangement expected for oxyanion stabilization of acylation and deacylation transition states.

The inhibitor forms an extended network of interactions with the active site, including two hydrogen bonds between TG2 and the peptide backbone, and hydrophobic interactions with the Phe residue of the peptide (Figure 4D and 4E). Additionally, the inhibitor exhibits good shape complementarity, burying more than 70% of its surface area upon complex formation. The penultimate (Pro) residue of the inhibitor assumes a *trans* configuration and orients the terminal Phe to bind in a deep hydrophobic pocket consisting of residues A304, L312, I313, F316, I331, and L420. Together, these features provide a clear structural basis for the strong specificity of TG2 for QxP(hydrophobic) signature sequences that are abundant in gluten peptides [20–22].

**Figure 5:**
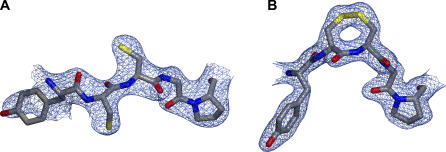
σ_A_ Weighted Electron Density Maps (2Fo-Fc) Contoured at 1σ in the Vicinity of Cys-370 and Cys-371 (A) In the GDP-bound structure [16], Cys-370 and Cys-371 are reduced. (B) In the inhibitor-bound structure, the cysteine residues form a vicinal disulfide bond, causing the intervening peptide bind to take on a *cis* configuration. (Carbon is indicated by grey, nitrogen by blue, oxygen by red, and sulfur by yellow.)

Surprisingly, our structure reveals the existence of a vicinal disulfide bond between two surface cysteine residues, C370 and C371 (Figure 5), which causes the intervening peptide bond to assume a *cis* configuration, distorting the peptide backbone. It has been shown that TG2 can be inactivated by the formation of a single intramolecular disulfide bond that does not involve the active cysteine [23,24], and more recently, that disulfide formation can interfere with the ability of TG2 to adopt a compact conformation upon incubation with GTP [25]. Because surface vicinal disulfides have been proposed to function as redox-activated conformational switches [26], it is possible that the C370–C371 disulfide bond underlies these redox features. In this context, we also note that the motif (F/Y)CCGP associated with this disulfide is conserved in TG1, TG2, TG4, TG5, and TG7, suggesting that these transglutaminases may also be sensitive to changes in redox potential.

### Solution Conformation of TG2

**Figure 6:**
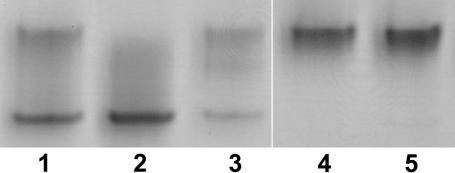
Nondenaturing PAGE Conformation Study of Recombinant Human TG2 TG2 was incubated with effectors, then subjected to electrophoresis. Lane 1: TG2 incubated without effectors. As purified, this sample assumes two conformational states. Extended dialysis of purified TG2 leads to virtual disappearance of the faster-migrating species, suggesting that in the absence of both GTP and calcium, the protein adopts an open-like conformation. Lane 2: incubation of TG2 with GTP and MgCl_2_, allosteric inhibitors of the enzyme, increases the relative abundance of the conformation with higher electrophoretic mobility. Lane 3: incubation of TG2 with CaCl_2_, the enzyme activator, reduces the relative abundance of the higher mobility conformer. The lower overall intensity of this lane can be explained by oligomerization, as evidenced by multiple bands of lower electrophoretic mobility (unpublished data). Lane 4: active-site–inhibited TG2 incubated without effectors assumes a conformation with the lower electrophoretic mobility. Lane 5: incubation of active-site–inhibited TG2 with GTP and MgCl_2_ does not cause a shift in mobility as it does for uninhibited TG2.

To investigate the physiological relevance of the open form of TG2 described above, we sought to characterize the closed (GTP-bound) and open (inhibitor-bound) conformations of TG2 in solution. We first verified that both forms were monomeric in solution, as determined by multi-angle laser light scattering coupled to size exclusion chromatography (SEC-MALLS; unpublished data). Next, we measured the radii of hydration of inhibitor-bound and GTP-bound TG2 using quasielastic light scattering and compared them to values calculated from the representative crystal structures (Table 1) [27]. In both cases, the measured values were within experimental error of the calculated values, confirming that the conformation of inhibitor-bound TG2 in solution resembles that observed in the crystal structure. Furthermore, the calculated radius of gyration of the inhibited TG2 matched a previous small-angle neutron scattering measurement of Ca^2+^-activated TG2 [28]. Lastly, native gel electrophoresis revealed that the mobility of inhibited TG2 was identical to that of nucleotide-free TG2 (Figure 6). Taken together, these results suggest that the overall conformations of nucleotide-free and Ca^2+^-activated TG2 in solution are similar to that observed in the “open” inhibitor-bound structure.

**Table 1:**
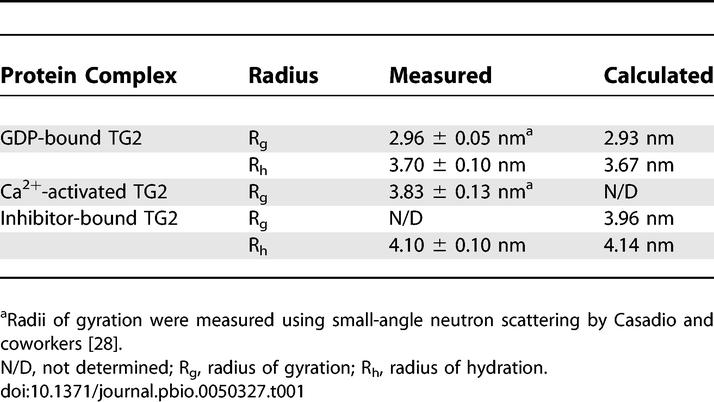
Measured Radii of Gyration and Hydration Are Compared to Calculated Values from the Crystal Structures

### T360 Mutations Cause TG2 to Favor Deamidation over Transamidation

Although our x-ray structure does not contain a bound nucleophilic substrate, the observed active-site geometry and steric constraints of protein transamidation suggest that the acyl-acceptor approaches from the open side of the active-site tunnel. T360, a residue that is conserved across all catalytically active members of the transglutaminase family, is located in front of the proposed acyl-acceptor tunnel entrance. To investigate the role of this residue, T360A and T360W mutants were generated by site-directed mutagenesis, and kinetics for deamidation and transamidation were measured. Although both activities were diminished, the mutants showed an increase in preference for deamidation with respect to transamidation compared to the wild-type enzyme. Moreover, the T360A mutant strongly favored deamidation, suggesting a possible role for this residue in transamidation catalysis (Table 2). To verify the essential catalytic role of the two tunnel-forming tryptophan residues, we constructed W241A and W332A mutants, both of which showed no detectable activity.

**Table 2:**
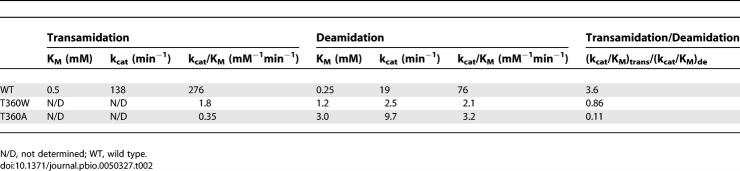
Kinetic Parameters of Wild-Type TG2, T360W, and T360A Mutants Using Ac-PQLPF-NH_2_ as the Acyl-Donor and Putrescine as the Acyl-Acceptor

## Discussion

Although conformational changes occur frequently in enzymes, observations of large conformational changes such as the one in this study are rare. Together, the x-ray crystal structures of the closed and open forms of TG2 provide fundamentally new insights into the biological dynamics of this ubiquitous, multifunctional, and tightly regulated protein. Moreover, as the prototypical structure of a catalytically activated transglutaminase, the crystal structure reported here also yields clues regarding the regulation of enzymatic activities of other members of the transglutaminase family. Additionally, as TG2 is the principal autoantigen in celiac sprue, the structure of human TG2 bound to a gluten peptide mimetic presents an opportunity to dissect this autoimmune response at a biochemical level.

### TG2 Biology

Although fibronectin crosslinking activity was the original biological function assigned to TG2, more-recent data suggest that the catalytically active form of mammalian TG2 is a relatively rare species, both in the intracellular and extracellular milieu. The absence of catalytic activity of intracellular TG2 is not surprising, given the allosteric effects of Ca^+2^ and GTP/GDP, and the demonstrated role of TG2 as a G-protein inside the cell [1]. Consistent with these observations, the active site of GDP-bound TG2 is in a closed conformation as a result of GDP binding between the first β-barrel domain and the catalytic domain (Figure S1). More surprising, however, are recent observations that (1) extracellular TG2 is catalytically inactive under ordinary physiological conditions (unpublished data), and (2) its ability to promote cell adhesion, spreading, migration, or differentiation is independent of catalytic activity [10,29]. The simplest explanation for these observations is that, like intracellular TG2, extracellular TG2 is also predominantly in a closed conformation. We have observed that catalytically inactive extracellular TG2 is transiently activated in response to innate immune signals such as exposure to poly(I:C), a potent ligand of the toll-like receptor TLR-3 (M. Siegel, P. Strnad, E. Watts, K. Choi, B. Jabri, M. B. Omary, C. Khosla, unpublished data). On the basis of these observations. we hypothesize that, in a normal stress-free environment, most extracellular TG2 is maintained in the closed conformation as a result of guanine nucleotide and/or integrin binding, despite relatively high extracellular Ca^2+^ concentrations. Physical or certain forms of chemical injury trigger rapid activation of TG2 into its catalytically active, open conformation.

### Mechanism and Regulation of Transglutaminases

Nine members of the mammalian transglutaminase family have been identified at the genetic level, seven of which have been characterized at the protein level (transglutaminases 1–5, factor XIIIa, and the catalytically inactive erythrocyte band 4.2). Although the regulation and substrate specificity varies significantly within this enzyme family, all active members share a high degree of sequence similarity, including strict conservation of key active-site residues such as the catalytic triad, W241, W332, and T360. In our structure, the dual hydrogen bonds between the inhibitor ketone and the indole of W241 and the backbone amide of the catalytic Cys (Figure 4D) are consistent with a catalytic mechanism involving oxyanion stabilization, a model that is supported by available biochemical data [30]. Interestingly, early kinetic analysis of both guinea pig liver TG2 and human factor XIIIa had led Folk to propose the transient formation of a tunnel in the active site during acylation [6]. Our results provide structural support for this hypothesis. Specifically, we propose that the bridging tryptophan residues and their corresponding loops (Figure 4C) separate, analogous to a drawbridge, in order to release the enzyme-bound transamidated product. On the basis of these observations, we predict that an analogous tunnel is also transiently formed between corresponding Trp residues in other transglutaminases during their catalytic cycle.

Notwithstanding their closely related catalytic mechanism, the activity of individual transglutaminase members is differentially regulated. For instance TG2, TG3, TG4, and TG5 [31–33], but not TG1 or factor XIIIa [34], are inhibited by GTP, and whereas TG3 requires cleavage at a loop between the catalytic core and the C-terminal β-barrels for full display of catalytic activity [35], cleavage at this loop leads to inactivation of TG2 and factor XIIIa. Activation of factor XIIIa is more intricate because the latent form is a heterotetramer consisting of two catalytic A subunits (factor XIIIa) and two regulatory B subunits. Activation involves thrombin cleavage of an N-terminal peptide on the A subunits, followed by dissociation of the tetrameric complex and a concerted conformational change in factor XIIIa [1]. In each case, a major conformational change such as that observed for human TG2 in this study is required for unmasking of the active-site residues and to allow access to protein substrates for crosslinking.

### Celiac Sprue Pathogenesis

The ability to visualize the structure of TG2 bound to a high-affinity gluten peptide analog has several potential consequences for our understanding of gluten-induced pathogenesis [36]. First, the atomic details of this covalent complex may facilitate a deeper understanding of the autoantibody response to TG2. If extracellular TG2 is predominantly in a closed conformation, then the open conformation described here is likely to expose self-epitopes that are ordinarily inaccessible to the immune system. In order for these “neo-epitopes” to trigger an autoantibody response via a hapten carrier-like model in celiac sprue, TG2–gluten adducts would have to be relatively stable. Enzymological analysis under conditions mimicking the gluten-fed small intestine has verified the unusual stability of the TG2–gluten peptide thioester adduct [20]. Thus, once extracellular TG2 in the celiac small intestinal mucosa is activated, the resulting TG2–gluten adducts may be sufficient to trigger an initial autoantibody response in HLA-DQ2–positive individuals. The question of course arises, how is extracellular TG2 activated in the celiac mucosa? As mentioned above, at least some innate immune signals, such as TLR-3 agonism, can induce TG2 activation. The expression of TLR-2, −3, and −4 is up-regulated in celiac mucosa, even in patients whose disease is in remission [37]. We therefore hypothesize that, either TG2 is constitutively in the open (catalytically active) conformation in celiac mucosa, or more likely, gluten triggers extracellular TG2 activation by interacting with certain up-regulated innate immune receptors in celiac mucosa. Notably, recent studies have suggested the occurrence of an innate immune response to gluten in celiac sprue patients [38–41].

Last but not least, the availability of high-resolution structures of both the GTP binding site and the active site of human TG2 facilitates inhibitor design for this pharmacologically significant protein. In particular, two distinct classes of pharmacological TG2 inhibitors are being explored: GTP analogs [42,43], which presumably stabilize the closed conformation, and active-site inhibitors, which trap the open conformation as shown in this study. Although both classes of inhibitors block TG2 catalytic activity, we predict that they will have different pharmacological effects. The ability to visualize protein–ligand interactions at both ligand binding sites should allow the rational design of inhibitors for pharmacological and therapeutic purposes.

## Materials and Methods

### Synthesis of inhibitor.

The peptide Ac-P(DON)LPF-NH_2_ was synthesized using a modified method described for the synthesis of similar products by Hausch et al. [44]. Briefly, Ac-PELPF-NH_2_ was synthesized via standard peptide synthesis techniques (Boc chemistry on 4-methylbenzhydryl amine resin). The N-terminus was acetylated using acetic anhydride (Sigma) on the resin, and the peptide was cleaved with triflic acid (Sigma). The resulting crude peptide was dried over potassium hydroxide in vacuo for several days. A total of 30 mg of crude peptide was dissolved in 4 ml of anhydrous THF (Acros); 1.6 equivalents N-methyl morpholine (Sigma) was added, followed by the dropwise addition of 1.2 equivalents iBu-COOCl (Sigma) on ice. The mixture was allowed to react for 5 min. Diazomethane was generated in situ by adding 1 M potassium hydroxide to a suspension of N-methyl-N-nitroso-p-toluenesulfonamide (Diazald; Sigma) in ethanol on ice. The evolved diazomethane was passed through a cannula and collected in 20 ml of extra-dry ether (Acros), using nitrogen as a carrier gas. The reaction mixture was added dropwise to the diazomethane solution and allowed to react for 30 min on ice, followed by 30 min at room temperature. The solvent was removed in vacuo, and the crude product was purified by C_18_ reverse-phase high performance liquid chromatography (HPLC) using a gradient of water + 0.1 M triethylamine bicarbonate (Fluka) and acetotnitrile + 0.1 M triethylamine bicarbonate. The product was verified by liquid chromatography–mass spectrometry (LC/MS) (M+H)^+^ = 667.3, and a yield of 17% quantified by UV_275_ absorbance.

### Protein expression and purification.

Site-directed mutagenesis of TG2 was performed using QuickChange mutagenesis (Stratagene) protocols and verified by sequencing. N-terminally His_6_-tagged wild-type and mutant human TG2 were expressed and purified as described in Piper et al. [20] with the minor modification that mutant proteins were induced at 13 °C.

### Preparative inhibition of TG2 by Ac-P(DON)LPF-NH_2_.

For large-scale preparation of inhibited TG2 for crystallization, freshly prepared TG2 (5 mg, no glycerol) was incubated in 3 ml of reaction buffer (20 mM Tris-HCl, 1 mM DTT, 1 mM EDTA, 150 mM NaCl [pH 7.2], and 10 mM CaCl_2_) with 2-mg Ac-P(DON)LPF-NH_2_ at room temperature for 30 min and then at 4 °C overnight. The protein was repurified by anion exchange chromatography, buffer exchanged, and concentrated as described for uninhibited TG2 [20]. Alternatively, lower concentrations of TG2 were used in the reaction buffer prior to anion exchange in some preparations. At higher concentrations of TG2, the protein precipitated from solution but subsequently redissolved upon dilution in anion exchange buffer. Each preparation yielded good-quality crystals, indicating that the protein was not denatured after the precipitation/redissolution process.

### Crystallization, data collection, and refinement.

Crystals of the TG2–inhibitor adduct were grown by the hanging-drop method at 25 °C using freshly prepared inhibited TG2 at 10 mg/ml with an equal volume of a precipitant buffer containing 100 mM HEPES (pH 7.25) and 1.25 M ammonium sulfate. Football-shaped crystals appeared in a few days and grew to about 150 μm × 150 μm × 300 μm in 1 wk. Crystals were soaked stepwise in mother liquor supplemented with 10%–20% glycerol and then flash frozen in liquid nitrogen. Diffraction data were collected at Advanced Light Source on beamline 8.3.1 and at Stanford Synchrotron Radiation Laboratory on beamline 11–1. The crystals belong to the space group P4_1_2_1_2 with unit cell dimensions a = 71.7 Å, b = 71.7 Å, c = 309.2 Å. All data were processed with DENZO and SCALEPACK [45].

The structure was solved by molecular replacement using human transglutaminase 2 structure (Protein Data Bank PDB code 1KV3) as a starting model. Although a molecular replacement solution was found using this structure, the β-barrel domains were clashing with symmetry-related mates. It was apparent that the inhibited TG2 undergoes large conformational change. The 1KV3 structure was therefore separated into four domains, which were searched independently in PHASER [46]. After the N-terminal and catalytic domains were located, their positions and orientations were refined in CNS. The positions and orientations of the two remaining β-barrels were sequentially located with PHASER, using fixed N-terminal and catalytic domains.

Alternate cycles of manual model building using the program O [47] and COOT [48]; positional and individual B-factor refinement with the program CNS [49] and REFMAC [50]; and addition of the inhibitor, water molecules, and sulfates to the model reduced the R and R-free to 22.8% and 26.6%, respectively, for all of the reflections (2.0-Å resolution). A total of 89.8% of the residues in TG2 are in the most favored regions of the Ramachandran plot, and 10.2% are in the additional allowed regions as calculated with PROCHECK [51]. The final model contains 5,522 atoms (5,188 protein, 47 ligand, 25 ion, and 262 waters) (Table S1). Residues 307–308, 319–327, 407–413, 462–471, and 684–687 are disordered in the structure and could not be built.

### SEC-MALLS.

A DAWN EOS (Wyatt Technology) with a K5 flow cell and a 690-nm wavelength laser was used in the light scattering experiments. Refractive index measurements were performed using an OPTILAB DSP instrument (Wyatt Technology) with a P10 cell. A value of 0.185 ml/g was assumed for the d*n*/d*c* ratio of the protein. Samples at approximately 3 mg/ml were passed over a Shodex-804 size exclusion column at 0.5 ml/min. Monomeric bovine serum albumin was used to normalize the detector responses. Astra software (Wyatt Technology) was used to analyze the light scattering data. Hydrodynamic radii were calculated with HYDROPRO [27].

### Kinetics.

The kinetic parameters of deamidation, transamidation, and inhibition were determined using a coupled enzyme assay and methods previously described [20,44,52]. Transamidation kinetics experiments were performed using 3 mM putresceine and varying glutamine donor concentrations.

### Native PAGE.

Native gel electrophoresis experiments were performed using a method similar to those previously described [25,53]. Briefly, TG2 or inhibited TG2 (2.5 μM) was incubated for 1 h at room temperature in preincubation buffer (75 mM imidazole, 0.5 mM EDTA, 5 mM DTT [pH 7.2]) with or without 500 μM GTP/1mM MgCl_2_ or 5 mM CaCl_2_. Laemmli native buffer was added, and 1.5 μg of protein was loaded onto a 4%–20% Tris-HCl ReadyGel (Bio-Rad) using Tris-glycine as the running buffer. For 75 min, 125 V was applied at 4 °C. The gel was stained with SimplyBlue SafeStain (Invitrogen) and destained with water.

## Supporting Information

Figure S1 Overlay of the Active Sites of TG2 in the GDP-Bound and Inhibitor-Bound Structures The inhibitor-bound structure of TG2 is shown in grey with the inhibitor in cyan. The equivalent in the GDP-bound structure is shown in blue. In the GDP-bound structure, the acyl-donor site is occupied by Y516, a residue located on a loop on β-barrel 1 (magenta) which is remote in the inhibitor-bound structure. The entire active site is buried by this β-barrel in the GDP-bound structure.
(2.0 MB TIF)

Table S1 Refinement and Model Statistics (25 KB DOC)

### Accession Numbers

The Protein Data Bank (PDB; http://www.rcsb.org/pdb) accession number/PDB ID for the GDP-bound human transglutaminase is 1KV3. Coordinates for human transglutaminase 2 inhibited with the active-site inhibitor Ac-P(DON)LPF-NH_2_ have been deposited in the Protein Data Bank under accession number 2Q3Z.

## Abbreviations

TG2: human transglutaminase 2
