# Supplementary material for: Transglutaminase 2 Undergoes a Large Conformational Change upon Activation
Source: PLoS Biol. 2007 Dec 18;5(12):e327. doi: 10.1371/journal.pbio.0050327 (PMC2140088; doi:10.1371/journal.pbio.0050327)
Supplement: Supplementary file 1 [file pbio.0050327.st001.doc]

**Table S1**

Refinement and Model Statistics

Space group	P4_1_2_1_2

# Cell dimensions

a, b, c (Å)	71.7, 71.7, 309.2

Molecules per asymmetric unit	1

Resolution (Å)	50.0 – 2.0

Measured reflections	336845

Unique reflections	53239

R_merge_	7.6 (33.9)

Completeness (%)	95.0 (78.5)

I / σ(I)	20 (2.5)

Number of protein atoms	5188

ligand atoms	47

ion atoms	25

water molecules	262

Overall	B factor value (Å^2^)	73.3

R_cryst_	22.8

R_free_	26.6

Root mean square deviation

Bonds	0.012

Angles	1.40

Ramachandran

most favored regions	89.8

additional allowed regions	10.2

generously allowed regions	0.0

disallowed regions	0.0
